# Vitamin C sensitizes BRAF^V600E^ thyroid cancer to PLX4032 via inhibiting the feedback activation of MAPK/ERK signal by PLX4032

**DOI:** 10.1186/s13046-021-01831-y

**Published:** 2021-01-19

**Authors:** Xi Su, Peng Li, Bin Han, Hao Jia, Qingzhuang Liang, Haichao Wang, Mengwei Gu, Jiaxuan Cai, Shaolei Li, Yaqi Zhou, Xin Yi, Wei Wei

**Affiliations:** 1 Department of Thyroid and Breast Surgery, Peking University Shenzhen Hospital, ShenZhen Peking University-The Hong Kong University of Science and Technology Medical Centre, No.1120, LianHua Road, FuTian district, Shenzhen, 518036 China; 2 Department of Merchandising, Walmart (China) Investment Co., Ltd, Shenzhen, China; 3 Department of Otorhinolaryngology, Peking University Shenzhen Hospital, ShenZhen Peking University-The Hong Kong University of Science and Technology Medical Centre, Shenzhen, China

**Keywords:** Thyroid Cancer, PLX4032, Vitamin C, MAPK/ERK pathway, BRAFV600E

## Abstract

**Background:**

BRAFV600E mutation is the most common mutation in thyroid cancer. It strongly activates MAPK/ERK pathway and indicates an invasive subtype of thyroid cancer. PLX4032 is a selective oral inhibitor of the BRAFV600 kinase although with limited effect in treating this panel of thyroid cancer, due to the feedback activation of MAPK/ERK as well as PI3K/AKT pathways. It was investigated that Vitamin C plays a positive role in inhibiting these pathways in thyroid cancer. However, whether Vitamin C could enhance the antitumor effect of PLX4032 remains largely unclear.

**Methods:**

The antitumor efficacy of combination therapy with PLX4032 and Vitamin C on BRAF^MT^ thyroid cancer cell was assessed by the MTT assay, EdU assay and colony formation, Chou-Talalay way was employed to analyze the synergistic effect. Flow cytometry were employed to assess cells’ apoptosis and cell cycle arrest in response to combination therapy. Xenograft models were used to test its in vivo antitumor activity. Western blot and IHC were applied to investigate the mechanism underlying synergistic effect.

**Results:**

PLX4032 or Vitamin C monotherapy was mildly effective in treating BRAF^MT^ thyroid cancer cell and xenografts model. The combination therapy significantly inhibited cancer cell proliferation and tumor growth in nude mice, and induced cell apoptosis and cell cycle arrest compared to either monotherapy. PLX4032 monotherapy induced feedback activation of MAPK/ERK as well as PI3K/AKT pathway; while combination therapy significantly relieved this feedback.

**Conclusion:**

Vitamin C promotes the antitumor effect of PLX4032 in BRAF^MT^ thyroid cancer cell and xenografts model via relieving the feedback activation of MAPK/ERK as well as PI3K/AKT pathway. PLX4032/Vitamin C combination may be a potential therapeutic approach to treat BRAF^MT^ thyroid cancer.

**Supplementary Information:**

The online version contains supplementary material available at 10.1186/s13046-021-01831-y.

## Background

The incidence of thyroid cancer, the most common endocrine malignancy, rapidly increased in recent years [1, 2]. Thyroid cancer is histologically classified of papillary thyroid cancer (PTC, 80–85%), follicular thyroid cancer (FTC, 10–15%), medullary thyroid cancer (MTC, 3–5%) and anaplastic thyroid cancer (ATC, < 2%) [3, 4]. Mitogen-activated protein kinase/extracellular signal-regulated protein kinase (MAPK/ERK) signal is particularly highly selected in PTC and a part of ATC [3, 5]. Though the new mechanisms underlying the formation of thyroid cancer were revealed recently [6–8], BRAFV600E mutation is the most common MAPK/ERK pathway-related genetic alteration in these types of thyroid cancer [3, 5, 9]. BRAF, as a member of the RAF family of serine/threonine protein kinases, is recruited and phosphorylating activated by RAS family. Active BRAF signals through MEK to activate MAPK/ERK pathway, which, in turn, induces a range of biochemical processes including cell differentiation, proliferation, growth, and apoptosis [10]. The residues’ glycine-rich segments G595-V600 hydrophobic interacts with segments G463-V470, keep the BRAF protein as an inactive conformation [9, 10]. The BRAFV600E mutation replaces V600 valine with E600 glutamic acid, a larger, charged side chain, causing the phosphomimic effect [9]. The phosphomimic effect breaks the hydrophobic interaction and destabilizes the inactive conformation, resulting in the constitutively active state and strongly activated MAPK/ERK signal [10, 11]. Therefore, targeting BRAFV600E could be the most promising treatment among patients with BRAF mutant (BRAF^MT^) thyroid cancer [12, 13].

Vemurafenib (PLX4032), a selective oral inhibitor of the BRAFV600E kinase, is associated with approximately 50% of response rate and improving survival among patients with BRAFV600E mutation–positive metastatic melanoma, which is another BRAFV600E mutation dominated malignancy [11, 14]. However, patients diagnosed with BRAFV600E-mutated thyroid cancers seldom benefit from PLX4032 due to the existence of MAPK/ERK as well as phosphatidylinositol 3-kinase/Protein Kinase B (PI3K/AKT) pathways feedback activation [15]. PLX4032 release the transcription repressor CTBP proteins from HER3 promoter and induce HER3 gene expression. Autocrine-secreted NRG1 binding to HER3, triggers HER3/HER2 heterodimerization and receptor phosphorylation, inducing PI3K and reactivating MAPK signaling, thus promoting resistance to growth inhibition [15]. Therefore, finding a new combining therapy to relieve this drug resistance and benefit these patients is of great significance.

Vitamin C, as an essential nutrient for humans, has been debated for years because of its controversial role in cancer therapy. More recently, it has been shown that vitamin C at pharmacologic plasma concentrations acquired intravenously can selectively kill the tumor cells but had little cytotoxic effect on normal cells; and these effect was mainly through a reactive oxygen species (ROS) dependent way [16–18]. These findings have inspired other studies to provide new insights into the function of combining therapy with vitamin C in treating different types of cancers [19–21]. Nevertheless, the function and the mechanism of combining therapy with vitamin C and PLX4032 in thyroid cancer has not been investigated yet. Although vitamin C shows effectiveness in treating the PLX4032-resistant melanoma cells; the authors did not explore the detailed mechanism [22]. Our previous study identified that Vitamin C could inhibit the MAPK/ERK as well as PI3K/AKT pathways [23]. We therefore hypothesize that the combination of Vitamin C and PLX4032 may synergistically induce the death of BRAF^MT^ thyroid cancer cell by relieving the feedback activation of MAPK/ERK as well as PI3K/AKT pathways.

In this study, we demonstrate that Vitamin C sensitizes BRAF^MT^ thyroid cancer cells to PLX4032. Vitamin C relieves the feedback activation of MAPK/ERK as well as PI3K/AKT pathways induced by PLX4032. As a result, combining Vitamin C with PLX4032 suppresses the malignant progression of thyroid cancer.

## Materials and methods

### Cell culture

Thyroid cancer cell lines 8305C and BCPAP were obtained from Chinese Academy of Sciences, Shanghai Institute of Biochemistry and Cell Biology. 8505C were obtained from Otwo Biotechnology Co., Ltd. All the cells were routinely cultured in RPMI 1640 medium (Gibco, Thermo Fisher Scientific) supplemented with 10% fetal bovine serum (Gibco, Thermo Fisher Scientific), 1% Non-Essential Amino Acids (Gibco, Thermo Fisher Scientific) and 1% Sodium Pyruvate (Gibco, Thermo Fisher Scientific) at 37 °C.

### Reagents

For in vitro study, BRAF inhibitor PLX4032 (Selleck Chemicals, Cat # S1267) and PLX4720 (Selleck Chemicals, Cat # S1152) were dissolved in dimethyl sulphoxide (DMSO), N-acetylcysteine (Sigma, Cat #A7250) and Vitamin C (Sigma, Cat # A4034) was dissolved in ddH2O, both in a high concentration. They were adding into the culture medium to dilute at the indicated concentration. For in vivo study, PLX4032 was dissolved in 0.5% hydroxypropyl methylcellulose (Sigma), and administered by oral gavage. Vitamin C was dissolved in ddH2O and administered by intraperitoneal injection at a total volume of 0.2 ml.

### Cell viability assay

Cells (3000 to 4000/well) were seeded in 96-well plates. After a 24-h’s culture, cells were pre-treated with indicated doses of Vitamin C or ddH2O for 6 h. Then the different doses of PLX4032 or PLX4720 were added to the medium for the indicated period. Next, the medium was refreshed and 20 μL of 5 mg/mL 3-(4,5-Dimethylthiazolyl-2)-2,5-diphenyltetrazolium bromide (MTT) was added into the medium and the plates were further incubated for 4 h, followed by adding 150 μl of DMSO. The plates were then read on a microplate reader using a test wavelength of 570 nm and a reference wavelength of 670 nm. All MTT assays were done in triplicate. IC50 values were calculated with the Reed-Muench method [24].

### Cell proliferation assay

5-Ethynyl-2′-deoxyuridine (EdU) assay kits were obtained from Solarbio, Beijing (Cat # CA1170). Briefly, cells (2–4 × 10^5^) were seeded in 12-well plates. After a 24-h’s treatment with 6 μM PLX4032 or 0.25 Mm vitamin C, individually or in combination, the medium was changed into 50uM EdU containing medium to further culture for 2 h. Then the cells were fixed with 4% paraformaldehyde and washed with PBS, followed by dyeing with Hoechst33342. The images were obtained with a fluorescent inverted microscope (Leica, Wetzlar, Germany, Cat # DMI8), and color mergence was performed using ImageJ image software (ImageJ version 1.44p, NIH, MD). The percent of positive staining cells were calculated from five random views.

### Colony formation assay

Monolayer culture was performed to measure colony formation. Thyroid cancer cells were seeded into 12-well plate at a concentration of 3000 cells per well, and cultured with various doses of Vitamin C and PLX4032 or PLX4720, individually or in combination. The medium was refreshed every 72 h. After 10–14 days of cultivation, colonies were fixed with 4% paraformaldehyde, and then washed with PBS and stained with a crystal violet solution. Each assay was carried out in triplicate.

### Cell apoptosis assay

Cells were seeded at a concentration of 60–70% in 6 well plate, and cultured with indicated doses of Vitamin C (0.25 mM) and PLX4032 (6 μM), individually or in combination (Vitamin C 0.25 mM and PLX4032 4 μM) for 48 h. All the cells were collected by centrifuged followed by washed twice with PBS and stained with Annexin V-FITC/PI Apoptosis Detection Kit (Roche Applied Science) according to the manufacturer’s protocol. Apoptotic cells were measured by flow cytometer (BD Biosciences, NJ). Each experiment was carried out in triplicate.

### Cell cycle analysis

Cells were seeded at a concentration of 40% in 6 well plate and serum starved for 24 h. After individually (Vitamin C 0.25 mM or PLX4032 6 μM) or in combination co-culture with 0.25 mM Vitamin C and 4 μM PLX4032 for 48 h, the cells were washed twice by PBS and fixed in 70% ethanol on ice for 30 min. Cells were then stained with PI solution (50 μg/mL PI, 50 μg/mL RNase A, 0.1% Triton-X, 0.1 mM EDTA). Cell cycles were analyzed based on DNA content using a flow cytometer (BD Biosciences, NJ).

### Animal studies

8305C (5 × 10^6^) cell lines were injected subcutaneously into the right armpit region of 5-week-old female nude mice purchased from SLAC laboratory Animal Co., Ltd. (Shanghai, China) to establish xenograft mouse model. Mice were then randomly divided into four groups (5 mice each group) when tumor volume grew to 20–30 mm^3^. PLX4032(50 mg per kg of body weight, once a day) and Vitamin C (3 g per kg of body weight, once a day) was administered individually or in combination. The treatment was administered for 2 weeks, body weight and tumor volume were measured with calipers every other day (Volume = width×length×width/2). Mice were sacrificed via cervical dislocation. Tumors were collected and weighted 5 h after the final dose of drugs. Animal experiments were approved by The Animal Experimental Center of Peking University Shenzhen Hospital. Ethical approval document of this research was provided at supplementary information, the approval number was 2020–328.

### Immunohistochemical (IHC) staining and H&E staining

Tumor tissues were embedded in paraffin, sectioned at 4 μm, then cell proliferation ability was assessed by quantification of Ki-67 staining (percentage of positive cells). In brief, antihuman Ki-67 antibody (BD Pharmingen, CAT 550609) and p-ERK (Cell Signaling Technology, CAT#4370) were 1:200–300 diluted, and immunostaining was done according to a standard protocol using DAB Substrate Kit (ZSGB-BIO). To ensure the comparability of immunohistochemical staining, a common reference standard was included as an internal or intra-assay control in each batch. Ki-67 protein expression was scored with 0, 1, 2, 3, which represent negative, weak positive, positive, and strong positive, respectively. Ki-67 protein expression scored with 1, 2, 3 were calculated as positive, the percent of positive staining cells were then calculated. ERK phosphorylation was quantitated by integral optical density (IOD) using Image-pro plus 6.0 (Media Cybernetics). Each stained section was evaluated under the same magnification, light brightness, and exposure intensity. To evaluate the effect of different treatments on different organs, we performed hematoxylin and eosin (H&E) staining of liver, kidney, and heart sections.

### Western blot analysis

The indicated cells were treated with PLX4032 4 μM and Vitamin C 0.5 mM, individually or in combination for 0,6,12,24 h. NAC 0.5 mM was added for 24 h in rescue experiment. Then cells were lysed in prechilled RIPA buffer containing protease inhibitors. Equal amounts of protein lysates were separated by SDS–PAGE and transferred onto PVDF membranes (Roche Diagnostics). The membranes were then incubated with primary antibodies. This was followed by incubation with species-specific HRP-conjugated secondary antibodies from ZSGB-BIO, and immunoblotting signals were visualized using the Western Bright ECL detection system (Tanon). The information of primary antibodies was listing as below: anti-tERK (Cell Signaling Technology, CAT #4695); anti-Pan-AKT (Bioworld Technology, CAT BS1810); anti-Actin (Santa Cruz CAT sc-1616).

### Statistical analysis

Synergy of the action of both compounds were statistical analyzed with Chou-Talalay method using ComboSyn Software (ComboSyn Inc. Paramus, NJ) [25, 26]. All the rest statistical analyses were performed with the SPSS statistical package (16.0, SPSS Inc. Chicago, IL). Unpaired student’s t test was used to compare the means of two groups of data. One-way analysis of variance (ANOVA) followed by Bonferroni’s multiple comparison test and Two-way ANOVA with Bonferroni post-test were used to compare differences between three or more groups. All values were expressed as the mean ± standard deviation (SD). *P* < 0.05 was considering statistically significant differences. Each experiment was carried out and calculated in triplicate.

## Results

### The combination of vitamin C and PLX4032 synergistically inhibited the growth of BRAF^MT^ thyroid cancer cells

**Fig. 1:**
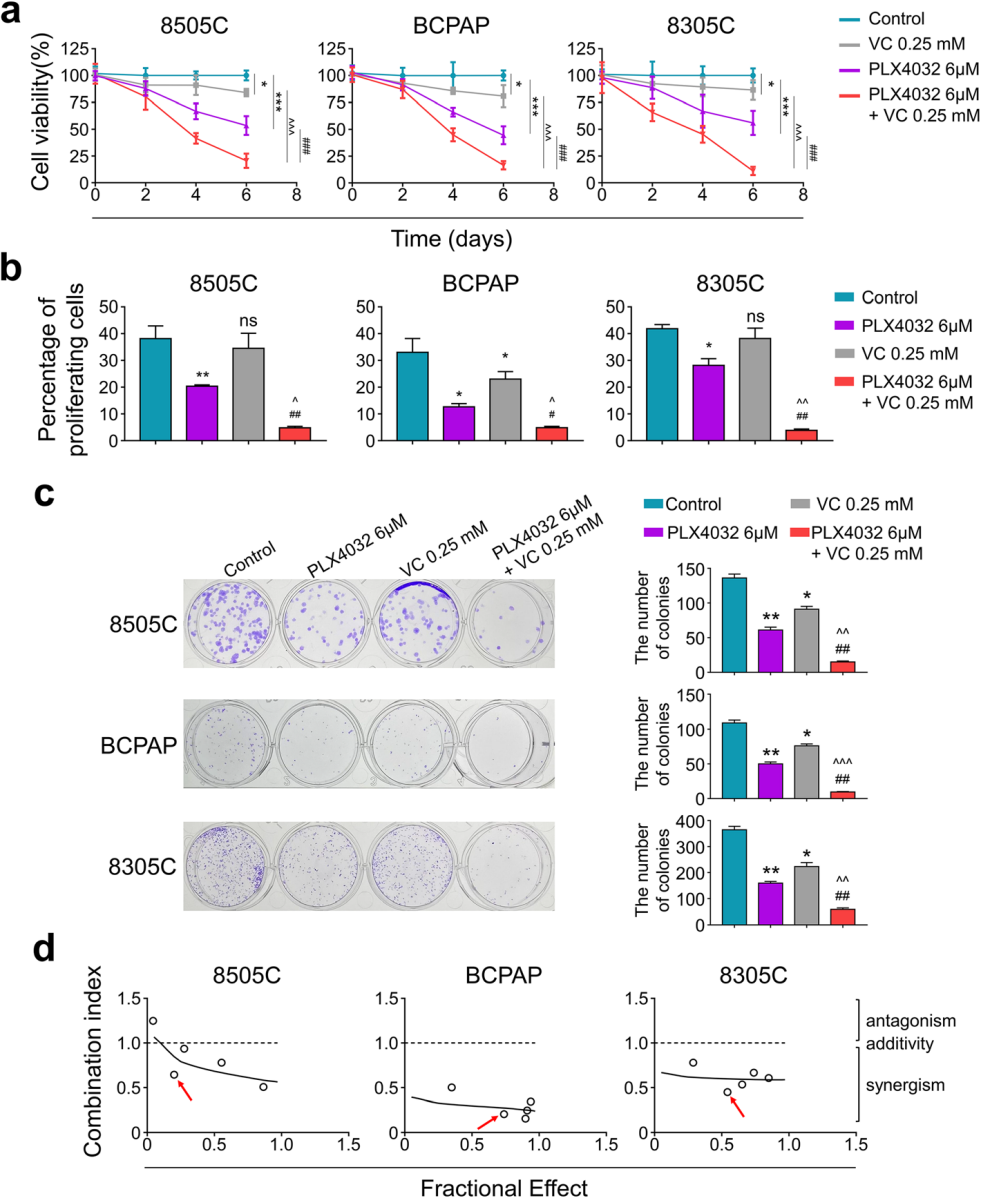
Combining Vitamin C (VC) and PLX4032 synergistic inhibited the proliferation of BRAF^MT^ thyroid cancer cells. **a** MTT assay was employed to evaluate cell viability after treating with indicating doses of VC and PLX4032 for 2–6 days. **b** Cell proliferation was evaluated with EdU assay after treating with indicating doses of VC and PLX4032 for 24 h. **c** colony formation assay was used to evaluate the proliferation inhibitory effect of combining therapy. **d** Analysis of the dose-effect relationship for PLX4032 (3-48 μM) and VC (0.1–2 mM) for the proliferative effect after 72 h of exposure in thyroid cancer cells, according to the Chou-Talalay dose-effect method. The combination index (CI) values were calculated: CI < 1, CI = 1, and CI > 1 represent synergism, additivity, and antagonism of these 2 agents, respectively. The “red arrow” represented the combination of VC (0.25 mM) and PLX4032 (6 μM). Data were presented as mean ± SD. ns, not significant; */^/#, *P* < 0.05; **/^^/##, *P* < 0.01; ***/^^^/###, *P* < 0.001. */ns: compared with control group; ^: compared with VC group; #: compared with PLX4032 group

We firstly performed the MTT assay to determine if combination of Vitamin C and PLX4032 could synergistically reduce the cell viability in thyroid cancer. As shown in Fig. 1a, the combination of Vitamin C (0.25 mM) and PLX4032 (6 μM) indicated a synergistic inhibitory effect on viability of cancer cells as compared with either Vitamin C (0.25 mM) or PLX4032 (6 μM) monotherapy. Next, we found combination of Vitamin C and PLX4032 also synergistic inhibited the proliferation (Fig. 1b and Supplemental Fig. 1) and colony formation (Fig. 1c) of BRAF^MT^ thyroid cancer cells 8505C, BCPAP and 8305C. To further confirm these findings, three cell lines were subjected to treatment with various concentrations of vitamin C and plx4032 for 72 h, individually or in combination. As can be seen in Fig. 1d, a synergistic effect was found in cells using the Chou-Talalay method. Besides, this synergistic inhibitory effect was also proved with another BRAF inhibitor-PLX4720 (Supplemental Fig. 2a-b), indicating vitamin C and BRAF inhibitor are acting synergistically. These results suggested that the combination of vitamin C and plx4032 may be an effective therapeutic strategy for BRAF^MT^ thyroid cancer cells.

**Fig. 2:**
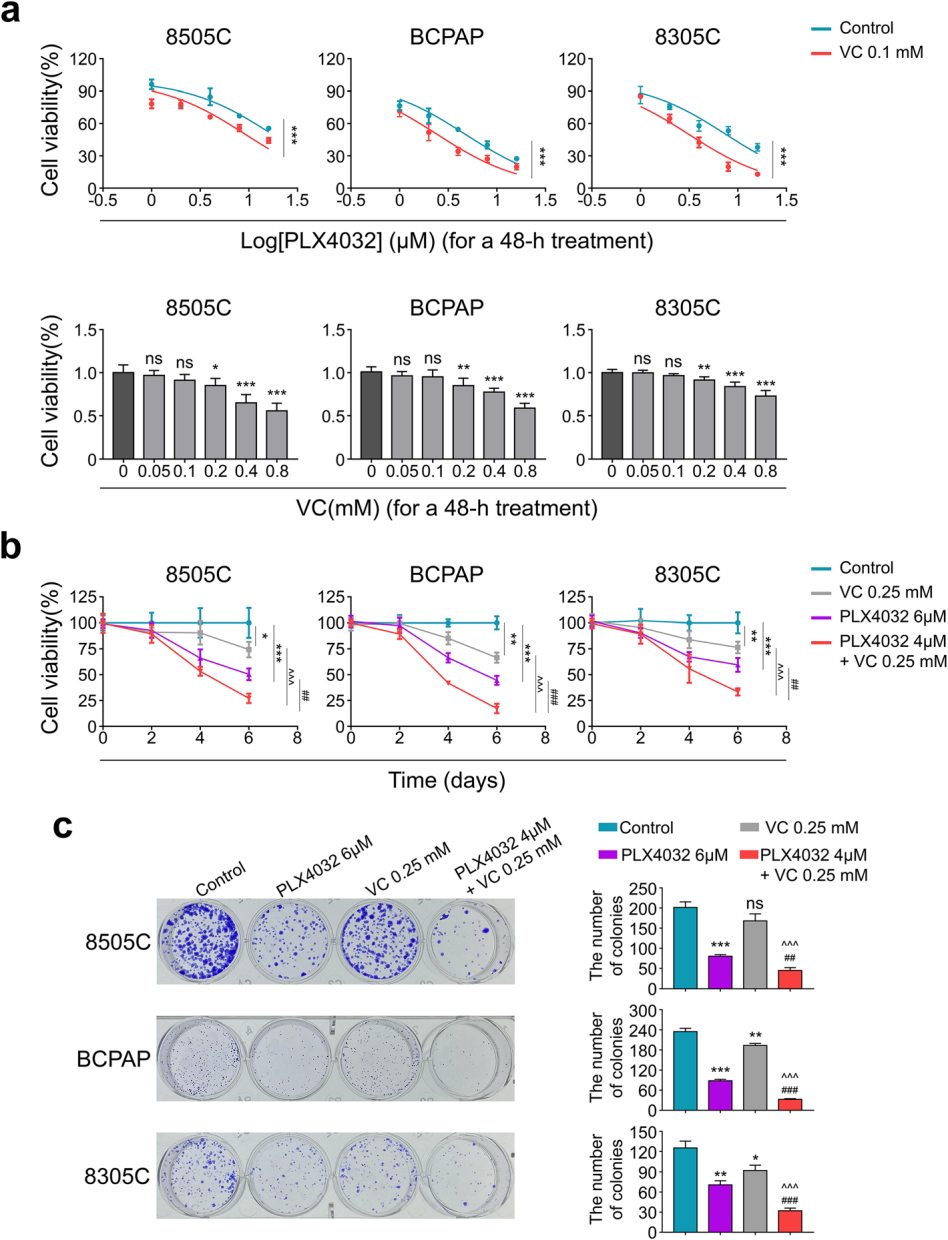
Vitamin C sensitized BRAF^MT^ thyroid cancer cells to PLX4032. **a** Cells were pre-treated with Vitamin C (VC) or vehicle for 6 h, followed by treating with different concentrations of PLX4032 (upper panels) or VC (lower panels) for 48 h, MTT assay was employed to evaluate cell viability. MTT assay (**b**) and colony formation assay (**c**) were used to evaluate the proliferation inhibitory effect of combining therapy. Data were presented as mean ± SD. ns, not significant; */^/#, *P* < 0.05; **/^^/##, *P* < 0.01; ***/^^^/###, *P* < 0.001. */ns: compared with control group; ^: compared with VC group; #: compared with PLX4032 group

Then, we performed the MTT assay to determine if Vitamin C could sensitize BRAF^MT^ thyroid cancer cells to PLX4032. As shown in Fig. 2a, combining with 0.1 mM vitamin C sensitized BRAF^MT^ thyroid cancer cells 8505C, BCPAP and 8305C to PLX4032 after 48 h’s treatment (upper panel), while single 0.1 mM Vitamin C didn’t affect the cell viability (lower panel). The calculated IC50 of PLX4032 were shown in Table 1. Moreover, the combination of Vitamin C (0.25 mM) and PLX4032 (4 μM) indicated a synergistic inhibitory effect on proliferation of cancer cells as compared with PLX4032 (6 μM) monotherapy (Fig. 2b). The observations were also proved by colony-forming assay (Fig. 2c). Taken all together, these results indicated that vitamin C potentiates the effects of PLX4032 against BRAF^MT^ thyroid cancer.

**Table 1:** IC50 of PLX4032

Cell lines	VC(0.1 mM)	IC50(μM)	95% CI of IC50(μM)
8505C	**–**	17.39	13.34–22.67
	**+**	9.21	7.50–11.31
BCPAP	**–**	4.75	3.95–5.72
	**+**	2.43	1.95–3.04
8305C	**–**	7.56	5.98–9.54
	**+**	3.14	2.61–3.79

The IC50 of PLX4032 was calculated in different cancer cells with or without the presence of Vitamin C

### Vitamin C sensitized thyroid cancer cells to PLX4032 induced apoptosis and cell cycle arrest

**Fig. 3:**
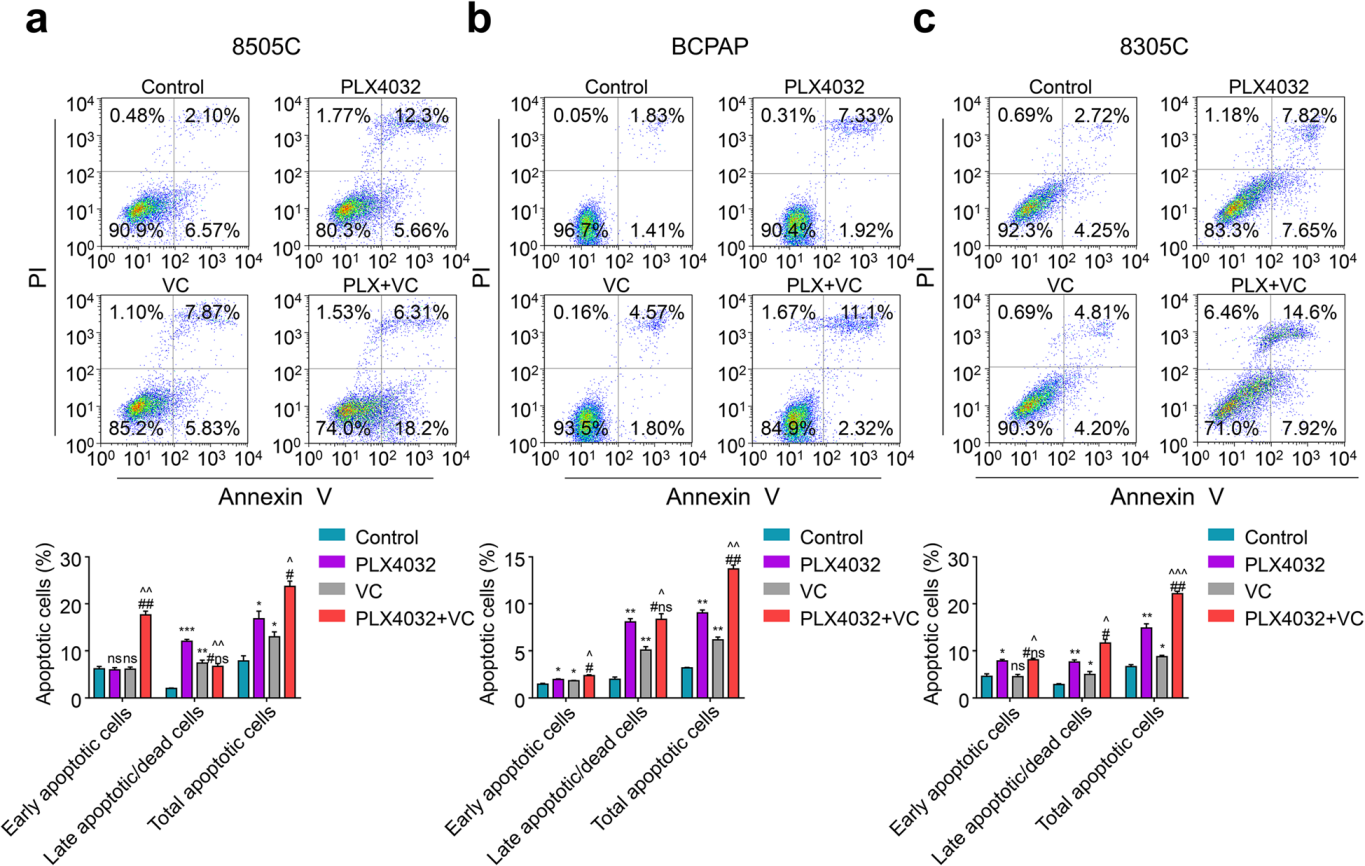
Vitamin C sensitized BRAF^MT^ thyroid cancer cells to PLX4032 induced apoptosis. **a** 8505C, (**b**) BCPAP, (**c**) 8305C cells were treated with 0.25 Mm Vitamin C (VC) or 6 μM PLX4032 individually, 0.25 Mm VC and 4 μM PLX4032 in combination for 48 h, apoptosis was measured by flow cytometry. Representative figures show the percentage of apoptotic cells (upper panels). Quantitative illustration of early apoptotic cells, late apoptotic/dead cells and total apoptotic cells were shown in down panels. Data were presented as mean ± SD. ns/#ns, not significant; */^/#, *P* < 0.05; **/^^/##, *P* < 0.01; ***/^^^, *P* < 0.001. */ns: compared with control group; ^: compared with VC group; #/#ns: compared with PLX4032 group

**Fig. 4:**
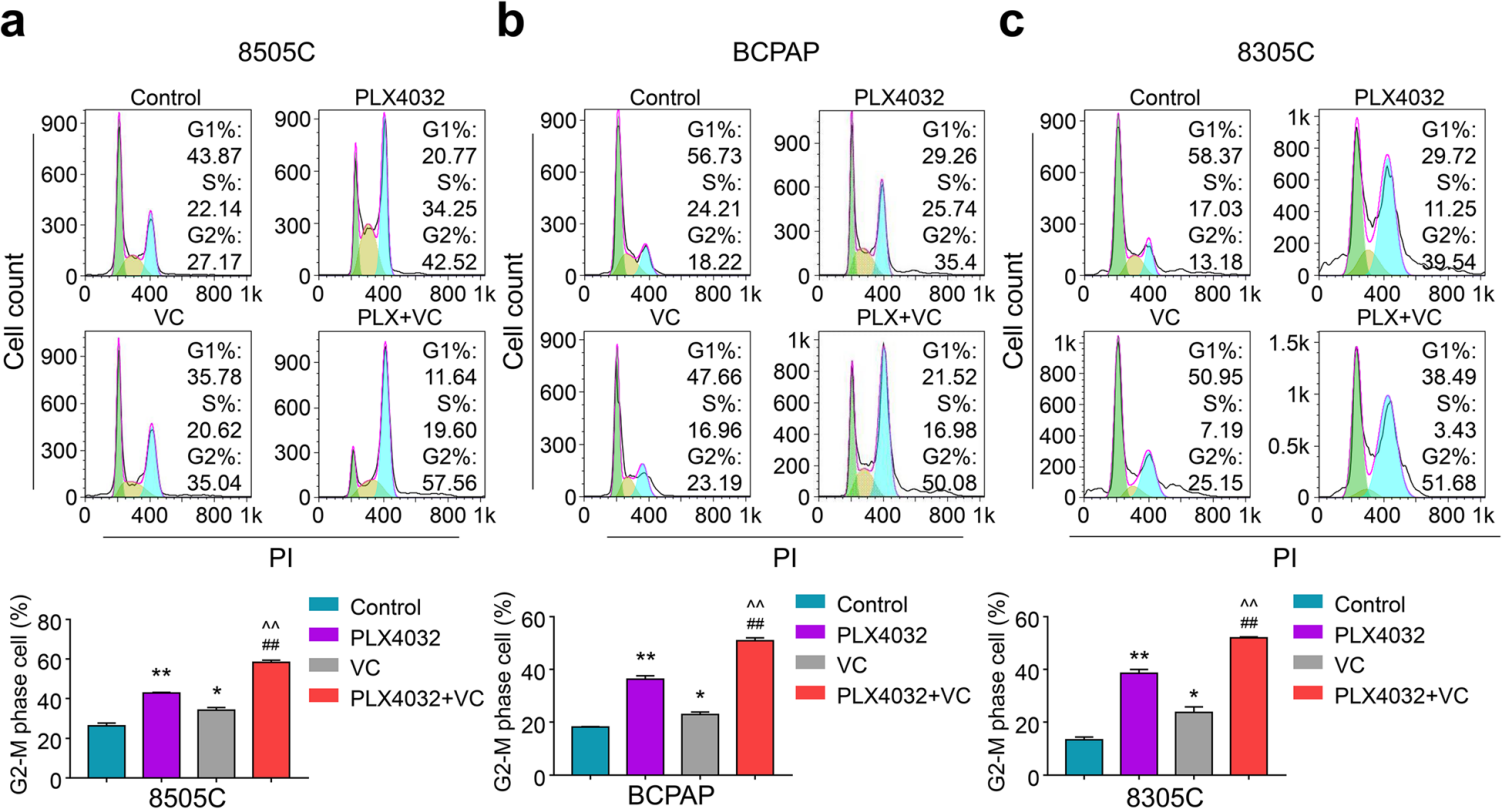
Vitamin C sensitized BRAF^MT^ thyroid cancer cells to PLX4032 induced cell cycle arrest. **a** 8505C, (**b**) BCPAP, (**c**) 8305C cells were treated with 0.25 Mm Vitamin C (VC) or 6 μM PLX4032 individually, 0.25 Mm VC and 4 μM PLX4032 in combination for 48 h, cell cycle arrest was measured by flow cytometry. Representative figures show the flow cytometric histograms (upper panels). Quantitative analysis of G2/M phase cells were shown in down panels. Data were presented as mean ± SD. *, *P <* 0.05; **/^^/##, *P* < 0.01. *: compared with control group; ^: compared with VC group; #: compared with PLX4032 group

Considering that inhibition of cancer cells is usually connected with cell cycle arrest and cell apoptosis [27, 28], we performed flow cytometry to investigate the contribution of combining Vitamin C with PLX4032 on cancer cells’ apoptosis and cell cycle arrest. In comparison with the vehicle control, both Vitamin C (0.25 mM) and PLX4032 (6 μM) caused apoptosis of BRAF^MT^ thyroid cancer cells (Fig. 3a-c). Moreover, Vitamin C (0.25 mM) potentiates the cytotoxicity induced by PLX4032 (4 μM) more than either monotherapy (Fig. 3a-c). Besides, the addition of Vitamin C to PLX4032 induced more G2/M phase cell cycle arrest among three BRAF^MT^ thyroid cancer cells compared to vehicle control or monotherapy (Fig. 4a-c).

### Vitamin C potentiates antitumor effect of PLX4032 in vivo

**Fig. 5:**
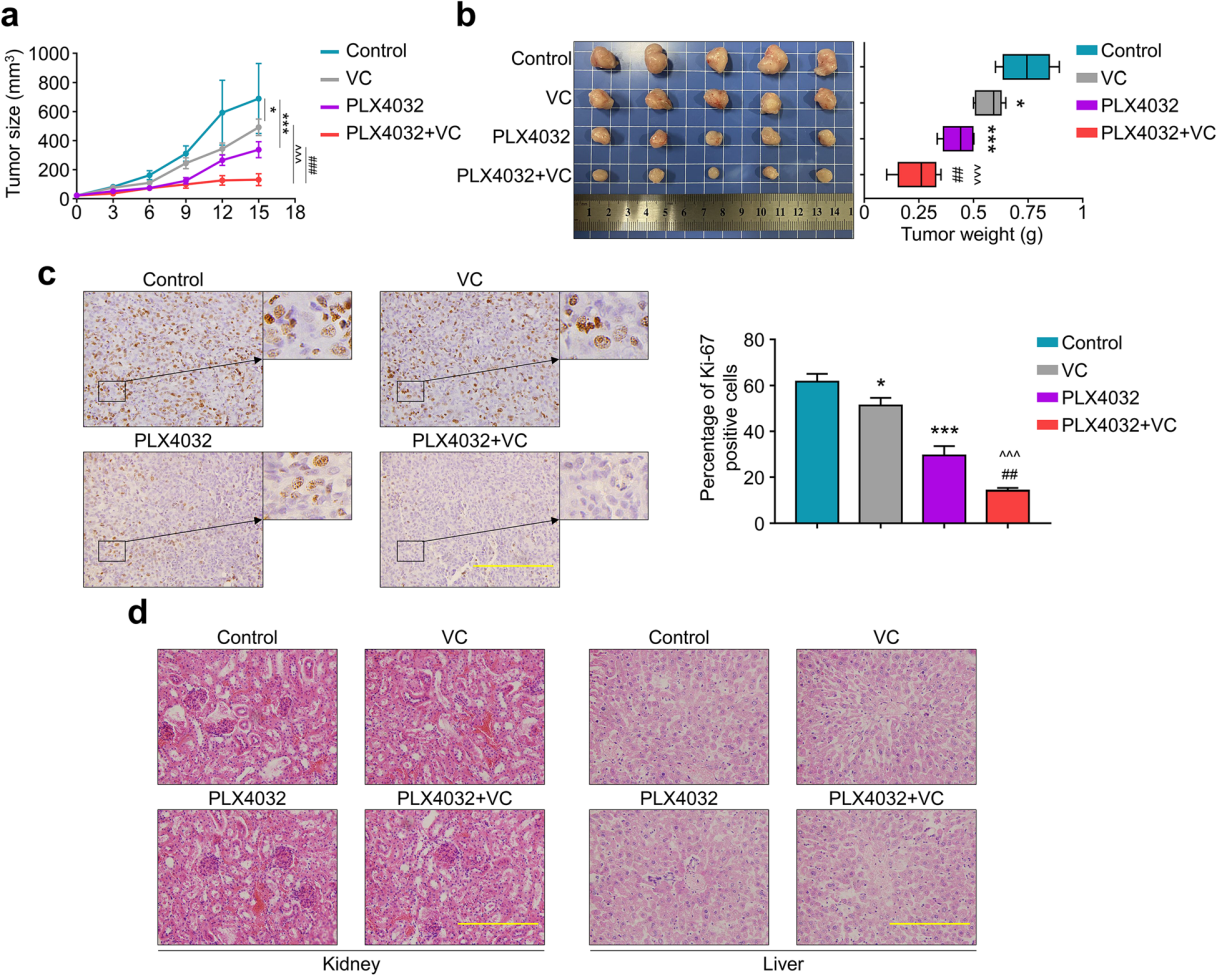
The combination of Vitamin C and PLX4032 synergistically inhibited the growth of BRAF^MT^ thyroid cancer cells in vivo. **a** The growth of xenografts tumors in different group were shown in the line graph. **b** Representative images show the isolated tumors from mice in different groups (left panel), and the bar chart illustrated the tumor weight. **c** The levels of Ki-67 proteins in the indicated xenograft tumors by IHC assay (left panels). Statistical analysis of the percentage of Ki-67 positive cells was shown in right panels. Scale bars, 200 μm. **d** Representative H&E stained slices of kidney and liver sections from the indicated mice. Scale bars, 200 μm. Data were presented as mean ± SD. VC: Vitamin C. *, *P <* 0.05; **/##, *P <* 0.01; ***/^^^/###, *P <* 0.001. *: compared with control group; ^: compared with VC group; #: compared with PLX4032 group

To further determine if Vitamin C potentiates antitumor effect of PLX4032 in vivo, BRAF^MT^ thyroid cancer cell 8305C were used to derive xenograft tumors in nude mice. As shown in Fig. 5a, xenograft tumors progressively grew in the control group, while PLX4032 or Vitamin C treatment reduced tumor growth (Fig. 5a). Besides, the combination of Vitamin C and PLX4032 induced a profound reduction in tumor growth, especially after 9-days’ administration, compared to either simple agent (Fig. 5a), on the other hand, the mice’s weight was not significantly decreased in each group (Supplemental Fig. 3). Moreover, the combination of Vitamin C and PLX4032 caused a significant reduction in both tumor volume and weight versus either monotherapy (Fig. 5b). To quantitatively assess the proliferation index in xenograft tumors, tumor sections were stained for Ki-67 expression [29]. As demonstrated in Fig. 5c, the PLX4032 or Vitamin C group showed lower Ki-67 staining as compared to the control, and the number of Ki-67 positive cells in tumors from combination group was lower than that in either monotherapy groups. On the other hand, histopathological evidences via H&E staining also verified that the combination of Vitamin C and PLX4032 do not cause more significant organ’s injury in mice than either monotherapy (Fig. 5d). As a result, our data demonstrated the efficacy and safety of the combination of PLX4032 and Vitamin C for BRAF^MT^ thyroid cancer treatment.

### Vitamin C potentiates antitumor effect of PLX4032 through relieving the feedback activation of MAPK/ERK as well as PI3K/AKT pathways in thyroid cancer cells

**Fig. 6:**
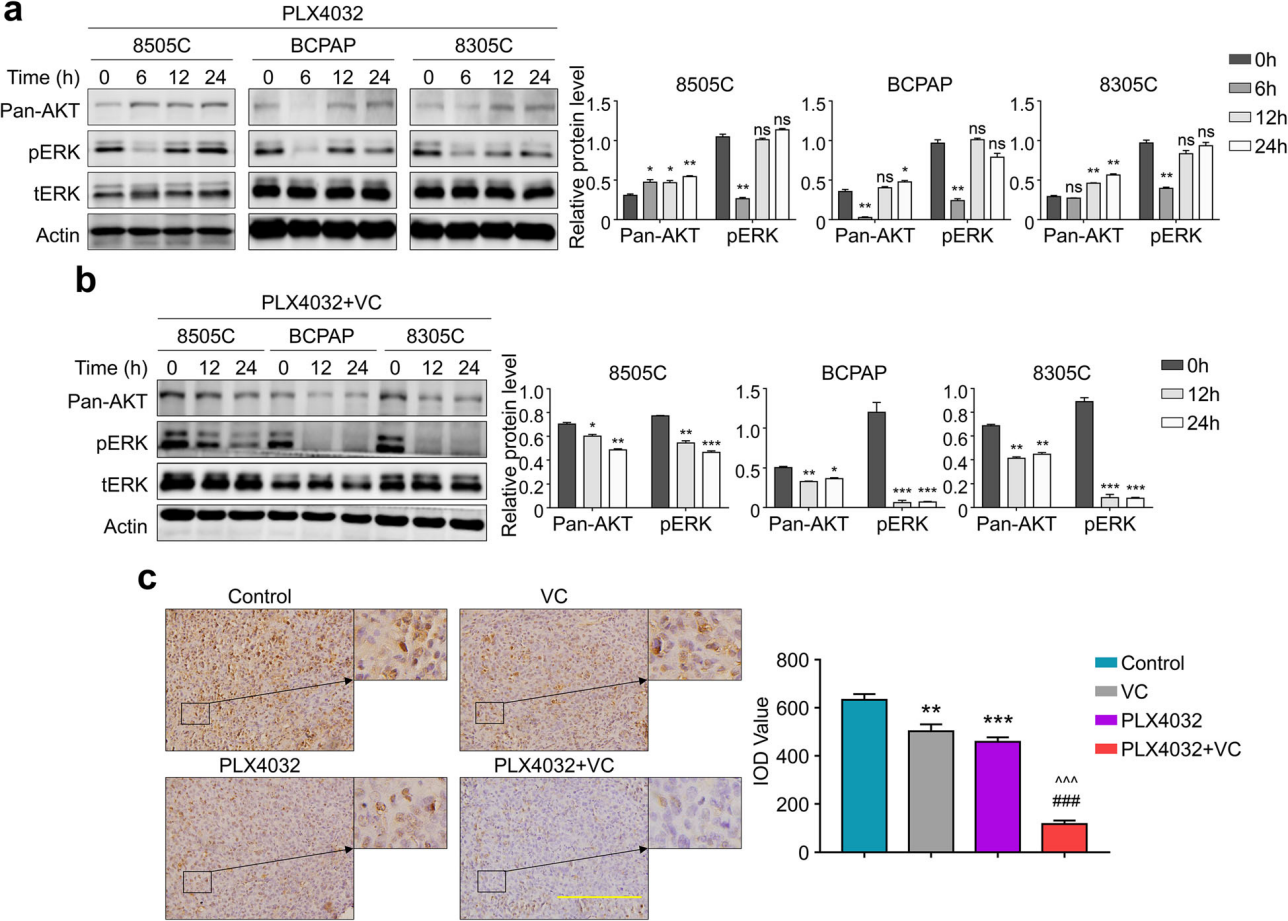
Vitamin C relieves the re-activation of MAPK/ERK and PI3K/AKT pathways caused by PLX4032. **a** The indicated cells were treated with PLX4032 4 μM for 0, 6, 12, 24 h, and cell lysates were then subjected to western blot analysis to determine the re-activation of MAPK/ERK as well as PI3K/AKT pathways (left panels). The normalized band intensity of Pan-AKT and pERK were shown in right panels. **b** The indicated cells were treated with PLX4032 4 μM and Vitamin C 0.5 mM (VC) for 0, 12, 24 h, and cell lysates were then subjected to western blot analysis to determine the re-activation of MAPK/ERK as well as PI3K/AKT pathways (left panels). The normalized band intensity of Pan-AKT and pERK were shown in right panels. β-Actin/tERK was used for normalizing Pan-AKT/pERK. **c** A comparison of the levels of p-ERK by IHC staining in xenografts slices. The expression levels were calculated with IOD value (right panels). Scale bars, 200 μm. Data were presented as mean ± SD. ns, not significant; *, *P <* 0.05; **, *P <* 0.01; ***/^^^/###, *P <* 0.001. */ns: compared with control group; ^: compared with VC group; #: compared with PLX4032 group

Previous study proved the using of PLX4032 will lead to the drug resistance in thyroid cancer cells mainly due to the re-activation of MAPK/ERK as well as PI3K/AKT pathway [15]. We thus firstly detected the level of ERK phosphorylation in different times after PLX4032 administration. As shown in Fig. 6a, after 6 h of adding the drug, PLX4032 significantly inhibited the ERK phosphorylation. However, after 12–24 h, the ERK phosphorylation rebounded, accompanying with the increased protein level of Pan-AKT (Fig. 6a). These results are consistent with previous studies that inhibition of MAPK/ERK signal by PLX4032 is transient in thyroid cancer [15]. Based on our previous observations [23], we assumed that Vitamin C may potentiate antitumor effect of PLX4032 by synergistically inhibiting the MAPK/ERK as well as PI3K/AKT pathways. As can be seen in Fig. 6b, combination of Vitamin C and PLX4032 tremendously inhibited the rebounding of ERK phosphorylation and the increasing of Pan-AKT after adding the drugs for 12–24 h. These results were further confirmed by the IHC staining of pERK in xenograft tumor sections, showing the combination of Vitamin C and PLX4032 greatly inhibited ERK phosphorylation than either monotherapy (Fig. 6c).

### Vitamin C relieving the feedback activation loops caused by PLX4032 in thyroid cancer cells is ROS dependent

**Fig. 7:**
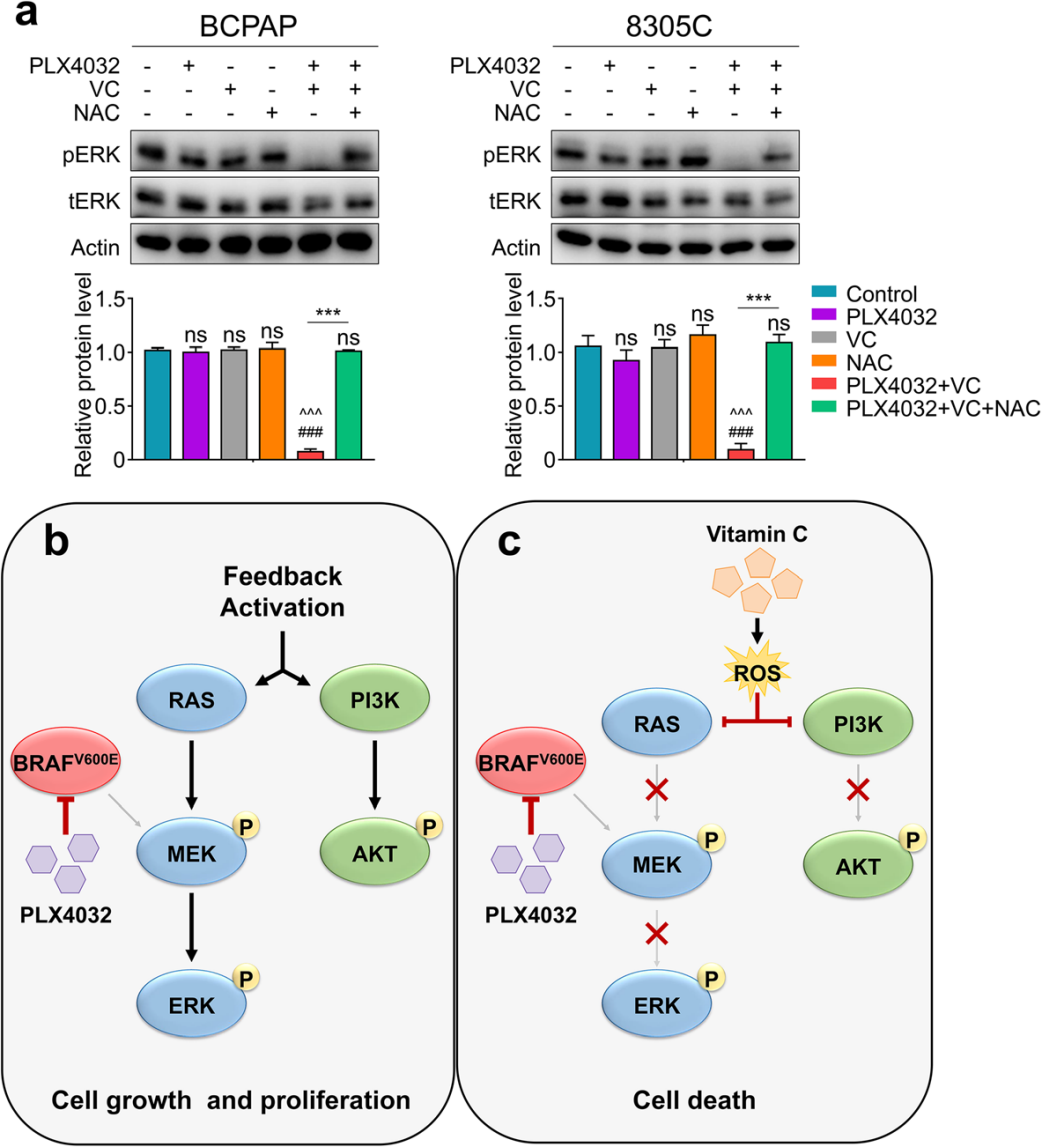
Vitamin C inhibit the feedback activation of MAPK/ERK pathways in a ROS dependent way. **a** The indicated cells were treated with PLX4032 4 μM, Vitamin C 0.5 mM (VC) with or without NAC 0.5 mM for 24 h, and cell lysates were then subjected to western blot analysis to determine the re-activation of MAPK/ERK pathways (upper panels). The normalized band intensity of Pan-AKT and pERK were shown in lower panels, tERK was used for normalizing pERK. **b** Treating BRAF^MT^ thyroid cancer with simple PLX4032 will induce a feedback activation of MAPK/ERK as well as PI3K/AKT pathways, leading to the drug resistance and cell survival. **c** When we combined Vitamin C and PLX4032, the feedback activation was inhibited by Vitamin C due to the accumulation of ROS, leading to the death of cancer cells. Data were presented as mean ± SD. ns, not significant; ***/^^^/###, *P <* 0.001. ns: compared with control group; *: compared withPLX4032 + VC group; ^: compared with VC group; #: compared with PLX4032 group

Previous research have shown that the tumor inhibitory function induced by Vitamin C was ROS dependent [16, 23], so we use NAC, a ROS eraser, to perform rescue experiment to find out whether it could abolish the decrease of ERK phosphorylation, which was induced by combining therapy. As demonstrated in Fig. 7a, the combination of Vitamin C and PLX4032 inhibited the rebounding of ERK phosphorylation after adding the drugs for 24 h, while NAC supplementation significantly weakened these effects. Based on the above observations, we propose a simple model to illustrate the mechanism of Vitamin C promoting the tumor inhibitory effect of PLX4032 in BRAF^MT^ thyroid cancer. To be short, treating BRAF^MT^ thyroid cancer with simple PLX4032 will induce a feedback activation of MAPK/ERK as well as PI3K/AKT pathways, leading to the drug resistance and cell survival (Fig. 7b). When we combined Vitamin C and PLX4032, the feedback activation was inhibited by Vitamin C due to the accumulation of ROS, leading to the death of cancer cells (Fig. 7c).

## Discussion

BRAFV600E mutation is recognized as the driving pathogenesis of most PTC and some ATCs, and thyroid-specific knock-in of BRAFV600E will induce an aggressive PTC [3, 30, 31]. This make BRAFV600E a prognostic molecular marker and promising therapeutic target for thyroid cancer, although in clinical trials the selective BRAFV600E inhibitor PLX4032 were not as promising as expected [32, 33]. The MAPK/ERK signal axis rebounding after the application of BRAFV600E specific inhibitors plays the key role in drug resistance [15].

In this study, we demonstrated Vitamin C sensitizing BRAF^MT^ thyroid cancer cells to PLX4032. Combining PLX4032 with Vitamin C synergistically inhibited cell proliferation, colony formation and tumorigenic potential in nude mice, and induced cell apoptosis and cell cycle arrest in BRAF^MT^ thyroid cancer cells. Our data indicated this synergistic effect was triggered by Vitamin C relieving the feedback activation loops caused by PLX4032. These results pointed out that combining PLX4032 with Vitamin C is a promising therapy in treating BRAF^MT^ thyroid cancer.

PLX4032 was a selective BRAFV600E inhibitor which was proven of high response rate in patients with metastatic melanomas [34]. In contrast to the high efficacy in patients with metastatic melanomas, PLX4032 as a single agent demonstrated limited response in patients with BRAF^MT^ colorectal cancers, which was ascribed to the activation of EGF receptor signal [35, 36]. Single agent of PLX4032 shows minimal activity towards patients with progressive, BRAF mutant PTC [37]. It was further confirmed by our data that PLX4032 exhibits a slight inhibitory action on the malignant activity of BRAF^MT^ thyroid cancer cells. Recent studies have proved that using PLX4032 will result in the reactivation of MAPK/ERK signal as well as PI3K/AKT pathways, leading to drug resistance in thyroid cancer [15]. Supporting by our data, an increased phosphorylation of ERK as well as pan-AKT in BRAF^MT^ thyroid cancer cells were observed after treating them with PLX4032 for 12–24 h. This was further validated by the IHC of phosphorylated ERK in xenograft. These results revealed that reactivation of MAPK/ERK signal and PI3K/AKT pathways will relieve the function of PLX4032 in BRAF^MT^ thyroid cancer.

In recent years, Vitamin C was proved to be a promising and potential therapy for certain cancers including thyroid cancer [16, 17]. In this study, we also proved the antitumor activity of Vitamin C in different BRAF^MT^ thyroid cancer cells. A series of in vivo studies also supported the antitumor efficacy of vitamin C through parenteral administration [38, 39]. Our results show that Vitamin C could enhance antitumor activities of the selective BRAFV600E inhibitor PLX4032 in both in vitro and in vivo study, and the Vitamin C dosage could be easily achieved via intravenous administration [38, 40]. More importantly, Vitamin C has been proved to be able to enhance the effect of some traditional therapies in different cancers [41, 42]. Therefore, we attempted to reveal the mechanism underlying our combining therapy. Our previous study has reflected that Vitamin C killed thyroid cancer cells, especially with mutant BRAF, through a ROS dependent inhibition of MAPK/ERK and PI3K/AKT pathways [23]. We thus hypothesized the reason behind was the inhibition of feedback activation of MAPK/ERK and PI3K/AKT pathways. In line with our hypothesis, combination of Vitamin C and PLX4032 greatly relieves the feedback activation of MAPK/ERK signal. It should be noted that, time-dependent inhibition of tumor growth in Fig. 5a revealed there is no difference between PLX4032 group and the combining group during the first 9 days after drug administration; while the tumor volume increased more quickly afterwards. We then assumed that the start of the feedback activation of MAPK/ERK signal has led to the result, while the combining therapy could further inhibit MAPK/ERK signal but not the PLX4032 monotherapy. However, extensive clinical trials may be necessary to determine the safety and efficacy of the combining therapy towards thyroid cancer.

In summary, we found that Vitamin C sensitizes BRAF^MT^ thyroid cancer cells to PLX4032 via relieving the feedback activation loops of MAPK/ERK and PI3K/AKT pathways. These preliminary results indicated that the Vitamin C/PLX4032 combination therapy may be an encouraging treatment in the cure of BRAF^MT^ thyroid cancer.

## Conclusion

Vitamin C promotes the antitumor effect of PLX4032 and synergistically inhibited the growth of BRAF^MT^ thyroid cancer cell and xenografts model with PLX4032. It significantly relieves the feedback activation of MAPK/ERK as well as PI3K/AKT pathway induced by PLX4032. The PLX4032/Vitamin C combination may be a potential therapeutic approach for BRAF^MT^ thyroid cancer.

## Supplementary Information

**Additional file 1: Supplemental Fig. 1.** The representative views of EdU staining of (**a**) 8505C, (**b**) BCPAP and (**c**) 8305C cell lines after a 24-h’s treatment with 6 μM PLX4032 or 0.25 mM vitamin C, individually or in combination. Scale bars: 100 μm.

**Additional file 2: Supplemental Fig. 2.** Cells were treating with 1 μM PLX4720 or 0.25 mM VC for 48 h, individually or in combination. MTT assay (**b**) and colony formation assay (**c**) were used to evaluate the proliferation inhibitory effect of combining therapy. Data were presented as mean ± SD. ns, not significant; *, *P* < 0.05; **/^^/##, *P* < 0.01; ***/^^^/###, *P* < 0.001. */ns: compared with control group; ^: compared with VC group; #: compared with PLX4720 group.

**Additional file 3: Supplemental Fig. 3.** The body weight of the nude mice was measured every other day after drug administration.

**Additional file 4.** Ethical approval document of this research.

## Data Availability

Wei Wei and Xi Su had the full access to all the data in the study and had final responsibility for the decision to submit for publication. And we declared that materials described in the manuscript, including relevant data, will be freely available to any scientist wishing to use them after informing Wei Wei and Xi Su.

## Abbreviations

PTC: Papillary thyroid cancer
ATC: Anaplastic thyroid cancer
BRAF^MT^: BRAF mutant
IHC: Immunohistochemical
IOD: Integral optical density
ROS: Reactive oxygen species
MAPK/ERK: Mitogen-activated protein kinase/extracellular signal-regulated protein kinase
PI3K/AKT: Phosphatidylinositol 3-kinase/Protein Kinase B
VC: Vitamin C
