# Supplementary material for: Vitamin C sensitizes BRAFV600E thyroid cancer to PLX4032 via inhibiting the feedback activation of MAPK/ERK signal by PLX4032
Source: J Exp Clin Cancer Res. 2021 Jan 19;40:34. doi: 10.1186/s13046-021-01831-y (PMC7816401; doi:10.1186/s13046-021-01831-y)
Supplement: Supplementary file 1 — Additional file 1: Supplemental Fig. 1. The representative views of EdU staining of (a) 8505C, (b) BCPAP and (c) 8305C cell lines after a 24-h’s treatment with 6 μM PLX4032 or 0.25 mM vitamin C, individually or in combination. Scale bars: 100 μm. [file 13046_2021_1831_MOESM1_ESM.docx]

**
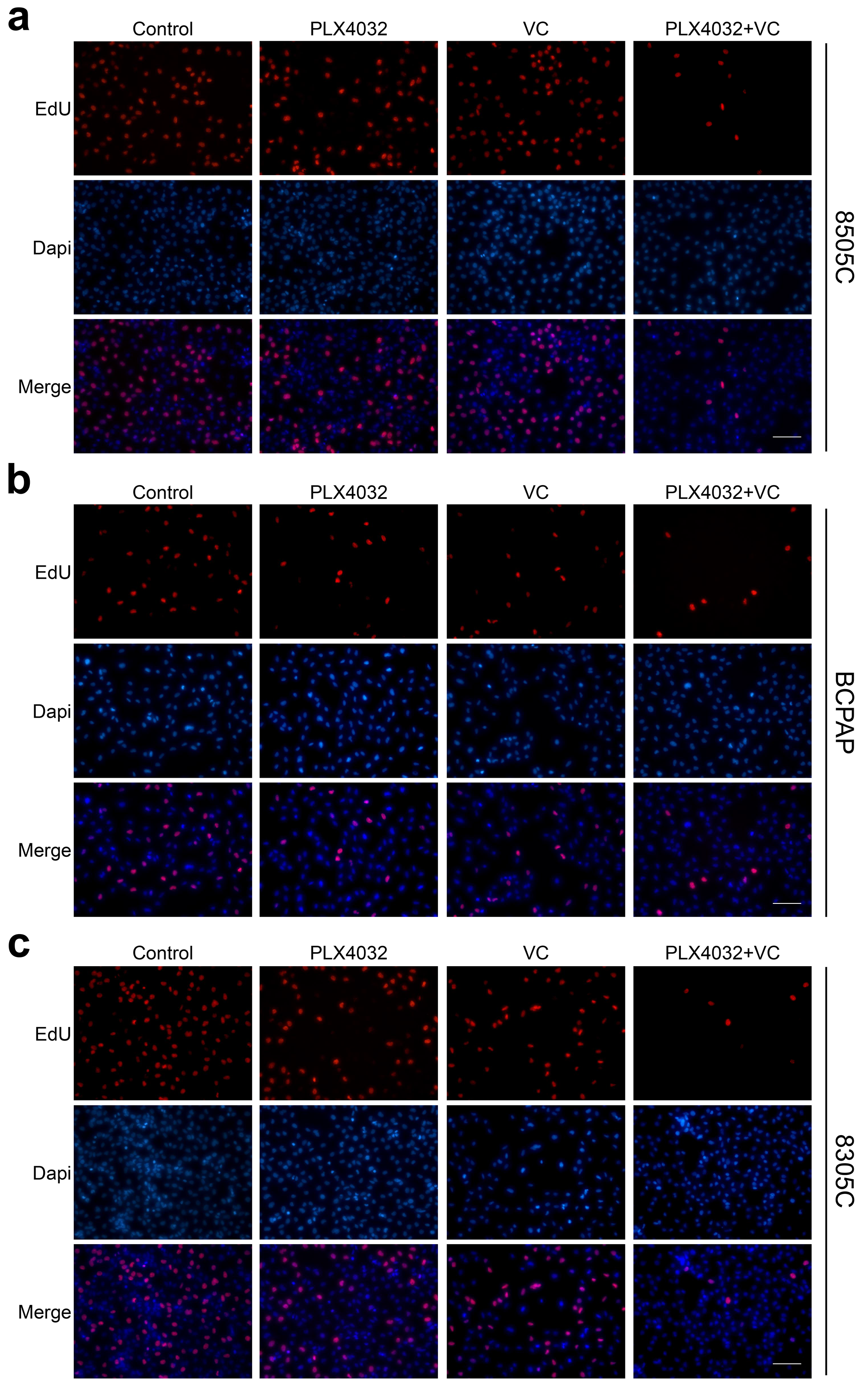
**

**Supplemental Fig. 1**. The representative views of EdU staining of (**a**) 8505C, (**b**) BCPAP and (**c**) 8305C cell lines after a 24-h’s treatment with 6μM PLX4032 or 0.25 mM vitamin C, individually or in combination. Scale bars: 100μm.
