# Supplementary material for: Vitamin C sensitizes BRAFV600E thyroid cancer to PLX4032 via inhibiting the feedback activation of MAPK/ERK signal by PLX4032
Source: J Exp Clin Cancer Res. 2021 Jan 19;40:34. doi: 10.1186/s13046-021-01831-y (PMC7816401; doi:10.1186/s13046-021-01831-y)
Supplement: Supplementary file 2 — Additional file 2: Supplemental Fig. 2. Cells were treating with 1 μM PLX4720 or 0.25 mM VC for 48 h, individually or in combination. MTT assay (b) and colony formation assay (c) were used to evaluate the proliferation inhibitory effect of combining therapy. Data were presented as mean ± SD. ns, not significant; *, P < 0.05; **/^^/##, P < 0.01; ***/^^^/###, P < 0.001. */ns: compared with control group; ^: compared with VC group; #: compared with PLX4720 group. [file 13046_2021_1831_MOESM2_ESM.docx]

**
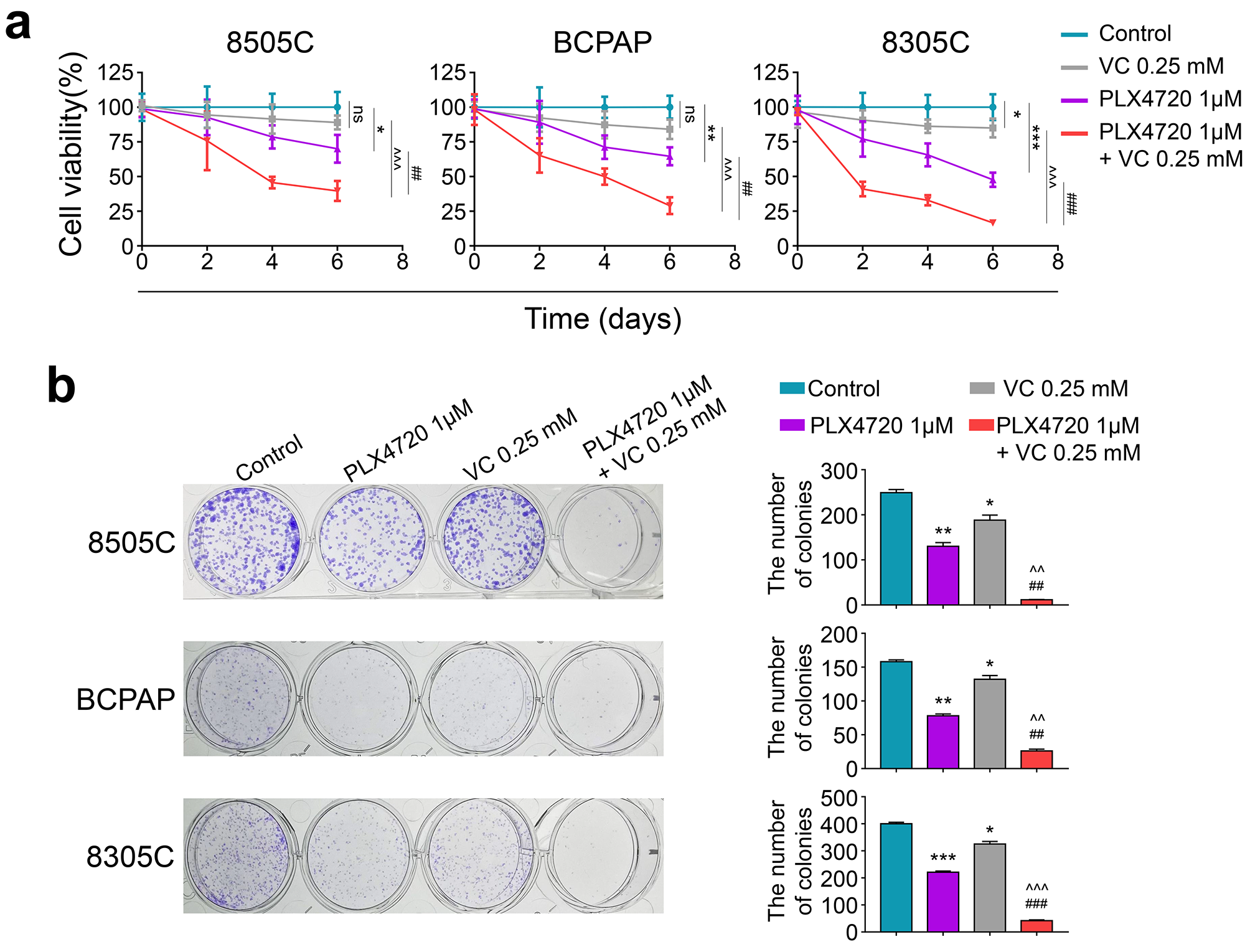
**

**Supplemental Fig. 2**. Cells were treating with 1μM PLX4720 or 0.25 mM VC for 48 h, individually or in combination. MTT assay (**a**) and colony formation assay (**b**) were used to evaluate the proliferation inhibitory effect of combining therapy. Data were presented as mean ± SD. ns, not significant; *, P < 0.05; **/^^/##, P < 0.01; ***/^^^/###, P < 0.001. */ns: compared with control group; ^: compared with VC group; #: compared with PLX4720 group.
