# Supplementary material for: Vitamin C sensitizes BRAFV600E thyroid cancer to PLX4032 via inhibiting the feedback activation of MAPK/ERK signal by PLX4032
Source: J Exp Clin Cancer Res. 2021 Jan 19;40:34. doi: 10.1186/s13046-021-01831-y (PMC7816401; doi:10.1186/s13046-021-01831-y)
Supplement: Supplementary file 3 — Additional file 3: Supplemental Fig. 3. The body weight of the nude mice was measured every other day after drug administration. [file 13046_2021_1831_MOESM3_ESM.docx]

**
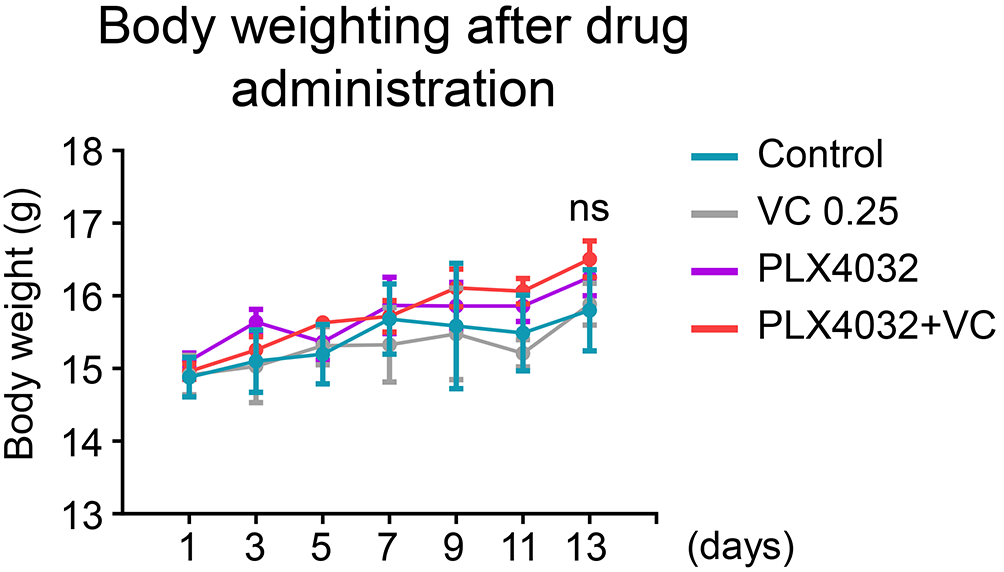
**

**Supplemental Fig. 3**. The body weight of the nude mice was measured every other day after drug administration.
