# Supplementary material for: Vitamin C sensitizes BRAFV600E thyroid cancer to PLX4032 via inhibiting the feedback activation of MAPK/ERK signal by PLX4032
Source: J Exp Clin Cancer Res. 2021 Jan 19;40:34. doi: 10.1186/s13046-021-01831-y (PMC7816401; doi:10.1186/s13046-021-01831-y)
Supplement: Supplementary file 4 — Additional file 4. Ethical approval document of this research. [file 13046_2021_1831_MOESM4_ESM.pdf]

实验动物福利伦理审查结果表

2020 年 11 月 05 日

实验编号	2020-328	项目执行人	韦伟
项目名称	维生素 C 通过抑制 PLX4032 引起的 MAPK/ERK 通路负反馈激活来增强 PLX4032 治疗 BRAF 突变型甲状腺癌效果的机制研究		
实验动物名称	BALB/c-nu、雌性，4-5 周，SPF 级		
申请人单位	北京大学深圳医院		
拟使用动物品系	BALB/c-nu	拟使用动物数量	32
审查类别	科研项目申报		
审查依据	1. 该项目是否必须使用实验动物进行实验，是否有其它替代方式；<br>2. 表中所填实验相关人员资格和实验相关单位是否合适；<br>3. 表中所填实验所用动物是否通过改良设计方案或高质量动物来减少所用动物数量；<br>4. 能否通过改进实验方法，调整实验观察指标，来优化实验动物，善待动物；<br>5. 实验设计及实验技术方法和实验动物使用数量是否合理；		
伦理委员会审查意见	符合深圳北京大学香港科技大学医学中心实验动物伦理学审查要求 建议批准伦理审查<br>符合伦理原则，同意申报		
伦理委员	张[illegible] 方[illegible]	主任委员	熊轶
实验动物福利伦理委员会（章）	深圳北京大学香港科技大学医学中心 实验动物伦理委员会	审查日期	2020 年 11 月 05 日

备注：在正式开展动物实验前，需要重新提交实验动物伦理审查申请。

扫描全能王 创建
